# The effects of intraoperative dexamethasone on left atrial function and postoperative atrial fibrillation in cardiac surgical patients

**DOI:** 10.1007/s12471-014-0638-5

**Published:** 2014-12-05

**Authors:** K. A. Jacob, J. M. Dieleman, H. M. Nathoe, D. van Osch, E. E. C. de Waal, M. J. Cramer, J. Kluin, D. van Dijk

**Affiliations:** 1 Department of Anesthesiology and Intensive Care, University Medical Center Utrecht, Mail Stop F.06.149, PO Box 85500, 3508 Utrecht, the Netherlands; 2 Department of Cardiothoracic Surgery, University Medical Center Utrecht, Utrecht, the Netherlands; 3 Department of Cardiology, University Medical Center Utrecht, Utrecht, the Netherlands

**Keywords:** Atrial fibrillation, Cardiac surgery, Left atrial ejection fraction, Left atrial dimension, Corticosteroids

## Abstract

Postoperative new-onset atrial fibrillation (PNAF) is very common after cardiac surgery and postoperative inflammation may contribute to PNAF by inducing atrial dysfunction. Corticosteroids reduce inflammation and may thus reduce atrial dysfunction and PNAF development. This study aimed to determine whether dexamethasone protects against left atrial dysfunction and PNAF in cardiac surgical patients. Cardiac surgical patients were randomised to a single dose of dexamethasone (1 mg.kg^−1^) or placebo after inducing anaesthesia. Transoesophageal echocardiography was performed in patients before and after surgery. Primary outcome was left atrial total ejection fraction (LA-TEF) after sternal closure; secondary outcomes included left atrial diameter and PNAF. 62 patients were included. Baseline characteristics were well balanced. Postoperative LA-TEF was 36.4 % in the dexamethasone group and 40.2 % in the placebo group (difference −3.8 %; 95 % confidence interval (CI) -9.0 to 1.4 %; *P* = 0.15). Postoperative left atrial diameter was 4.6 and 4.3 cm, respectively (difference 0.3; 95 % CI −0.2 to 0.7; *P* = 0.19). The incidence of PNAF was 30 % in the dexamethasone group and 39 % in the placebo group (*P* = 0.47). Intraoperative high-dose dexamethasone did not protect against postoperative left atrial dysfunction and did not reduce the risk of PNAF in cardiac surgical patients.

## Introduction

Postoperative new-onset atrial fibrillation (PNAF) is a complication occurring in 25–30 % of cardiac surgical patients. PNAF may lead to significant morbidity, including stroke, renal and respiratory failure, and is associated with an increased mortality [1–3].

Several preoperative echocardiographic findings have been implicated as predictors of PNAF. These predictive measures include increased left atrial (LA) volume and dysfunction [4–9]. A recent study showed that decreased LA total ejection fraction (TEF) was the most potent predictor of PNAF [10].

The underlying pathophysiological mechanisms are complex. Apart from pre-existing atrial dysfunction, the perioperative systemic inflammatory response may play a role in affecting atrial function, along with atrial myocardial injury and ischaemia [11–13]. The importance of inflammatory mechanisms is supported by several studies, demonstrating that atrial biopsies taken during cardiac surgery show marked oxidative damage and interstitial fibrosis [11, 13, 14].

The effects of administration of corticosteroids on the perioperative inflammatory response during cardiac surgery have been extensively investigated. Corticosteroids are thought to decrease the levels of serum inflammatory markers [15], reduce the risk of postoperative respiratory failure, and shorten the duration of postoperative intensive care unit and hospital stay [16, 17]. Furthermore, steroids may reduce the incidence of PNAF. Although the latter effect seems related to the attenuated inflammatory response [18], the exact mechanisms are unclear.

This study aimed to evaluate the effect of dexamethasone administration on postoperative LA dysfunction and dilatation. We hypothesised that high-dose dexamethasone increases LA-TEF, decreases postoperative LA diameter and protects against PNAF.

## Materials and methods

### Study design and patient selection

The DECS-PNAF study was a substudy of the Dexamethasone for Cardiac Surgery (DECS) study. It was a pre-planned and prospectively designed and performed substudy in which patients with a minimum age of 18 years, undergoing cardiac surgery, were enrolled in this multicentre randomised, double-blind, placebo-controlled study, comparing high-dose intravenous dexamethasone with placebo treatment (trial registration: ClinicalTrials.gov, number NCT00293592) [16]. DECS study patients in one of the participating centres, i.e. University Medical Centre Utrecht, who were scheduled for coronary artery bypass grafting (CABG), aortic valve surgery, or a combination of these procedures, without other concomitant valve surgery or other additional procedures, were eligible for inclusion in this DECS-PNAF study. Patients with either a history of atrial fibrillation, a contraindication for intraoperative transoesophageal echocardiography, or a preoperative cardiac rhythm other than sinus rhythm, were not eligible for inclusion.

The research ethics committee of the University Medical Center Utrecht approved the research protocol. All patients provided written informed consent before randomisation. The study was performed in accordance with the Medical Research Involving Human Subjects Act (WMO) and institutional regulations and guidelines.

### Intervention

Patients were randomised to receive either dexamethasone or placebo treatment. Dexamethasone (1 mg.kg^−1^ with a maximum of 100 mg) or placebo was administered as a single intravenous injection after induction of anaesthesia, but before commencement of cardiopulmonary bypass. Comprehensive information about the randomisation methods has been published previously [16].

### Primary and secondary outcomes

The primary endpoint of this study was the LA-TEF at the end of the surgical procedure. Secondary endpoints included LA diameter, LA area, maximum and minimum LA volume in the cardiac cycle, left atrial appendage (LAA) area and velocity and left pulmonary vein systolic/diastolic (S/D) ratio at the same time point. We also evaluated the occurrence of PNAF during the first 5 days after surgery.

### Data collection

Baseline patient characteristics and surgical characteristics were prospectively collected as part of the DECS study [16]. For the present substudy, patients underwent transoesophageal echocardiography after induction of anaesthesia, and after sternal closure at the end of the operation, while the patient was in a supine position. The required images were stored and analysed offline by two independent blinded observers, using the Philips XCELERA Cardiology image (Version 3.2) software package. All measurements were done twice and averaged.

LA diameter was measured from the posterior LA wall to the mitral annulus, at the end of ventricular systole in the mid-oesophageal long-axis view with the transducer array at ~120° [19, 20]. LA volume was assessed by the area-length method from both the mid-oesophageal four-chamber and two-chamber views, with LA size being optimised in both views. Biplane LA volume was calculated using Simpson’s method. The LA volume was measured at both the end of diastole (left atrial end-diastolic volume, being maximum LAV), and at the end of systole (left atrial end-systolic volume, being minimum LAV) [20]. LA-TEF was calculated using the following formula: (((LAVmax - LAVmin)/LAVmax) × 100 %). In patients in whom the entire dimensions or volume of the LA were not visible in the scan sector, measurements were traced to the edge of the field of view. The LAA was imaged in the mid-oesophageal two-chamber view. The LAA area was calculated using Simpson's method, at the end of ventricular systole, and peak velocity was measured by pulse wave Doppler, with the pulsed Doppler sample volume placed in the LAA cavity. Peak flow velocity in the left pulmonary vein was recorded and measured using a biphasic systolic and diastolic velocity wave. The S/D ratio was calculated afterwards.

The occurrence of PNAF was evaluated with continuous Holter monitoring (leads V1-V5) for the first 5 days postoperatively in all patients. The occurrence of PNAF was defined as the occurrence of any episode of atrial fibrillation or atrial flutter during any of these five postoperative days.

### Statistical analysis

Normally distributed continuous variables are presented as means with standard deviations (±SD). Data in both groups were compared using an independent *t*-test. Continuous data that were not normally distributed are presented as medians with interquartile range and were compared using a Mann–Whitney *U*-test. Dichotomous characteristic variables are presented as absolute numbers with percentages and were compared using a *χ* ^2^ test. We calculated mean differences with 95 % confidence intervals (CIs), for continuous and dichotomous outcomes, respectively. In addition to the analyses based on the randomisation to dexamethasone or placebo, we evaluated the association between LA-TEF and PNAF using an independent *t*-test. A *p*-value < 0.05 was considered significant in the analysis models. Statistical analysis was performed using IBM SPSS version 20.0 (SPSS Inc., Chicago, IL, USA).

## Results

**Fig. 1:**
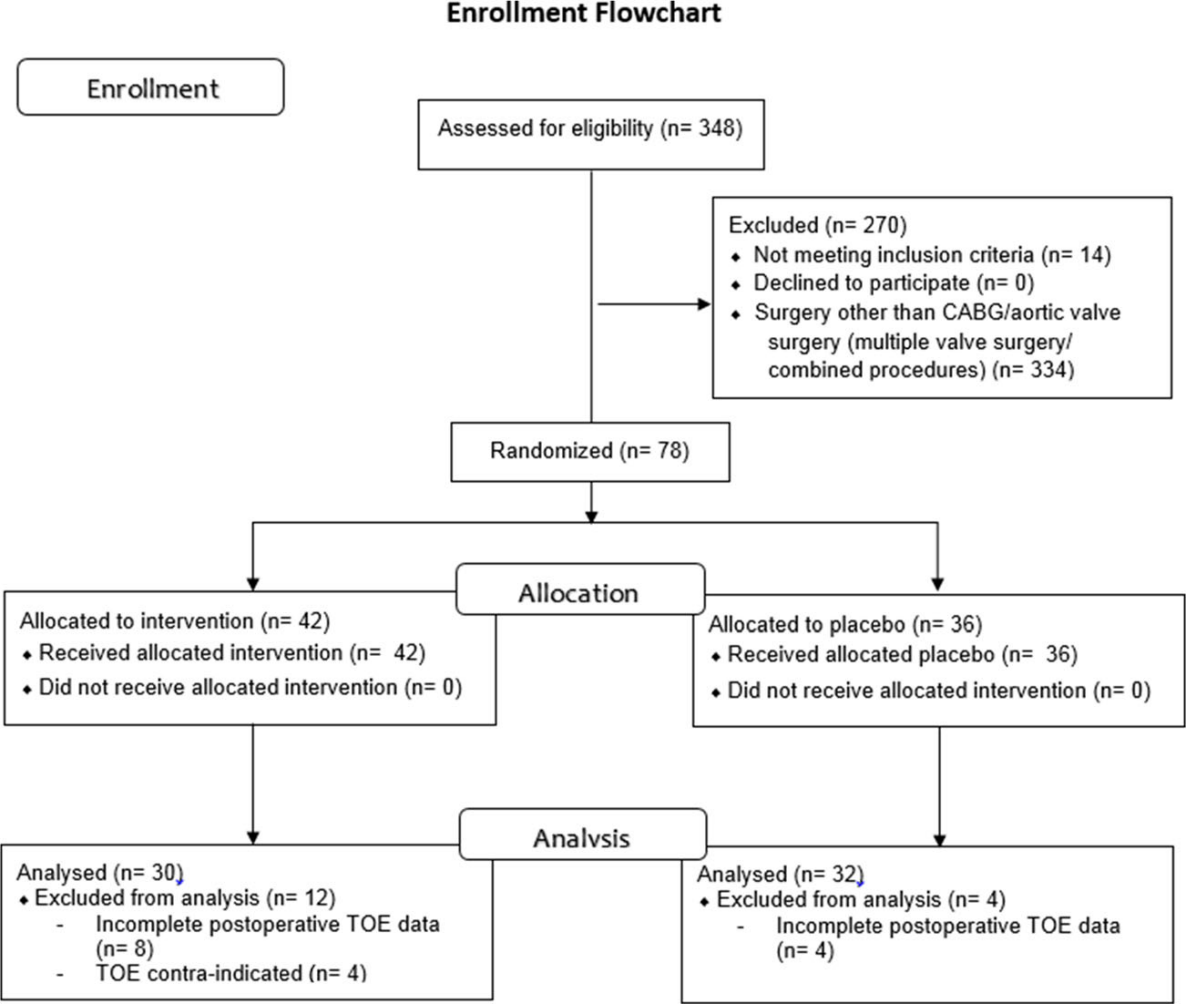
Enrolment flowchart. In the 62 patients included for postoperative analysis. *TOE*: transoesophageal echocardiography

The DECS-PNAF transoesophageal echocardiography substudy included 62 patients (Fig. 1). Patients in the two study groups were comparable with no statistical differences in baseline demographic, clinical, and surgical aspects (Table 1).

**Table 1:** Demographic, clinical, and surgical characteristics of the dexamethasone and placebo groups^α^

Characteristics	Dexamethasone *N* = 30	Placebo *N* = 32
Demographics		
Age, mean (SD), years	70.4 (9.1)	68.9 (9.0)
Male sex	24 (80)	23 (72)
BMI, mean (SD), kg/m^2^	27.96 (4.79)	26.83 (3.28)
BSA, mean (SD), m^2^, ^β^	2.04 (0.20)	1.97 (0.17)
Coexisting medical conditions		
Hypertension	18 (60)	20 (63)
Diabetes mellitus	7 (23)	3 (9)
Treatment for pulmonary disease	2 (7)	7 (22)
Peripheral vascular disease	5 (17)	8 (25)
Preoperative creatinine, mean (SD), μmol/l	89.3 (18.6)	92.8 (18.6)
Recent myocardial infarction	6 (20)	5 (16)
Left ventricular function, Good, ^γ^	21 (70)	21 (66)
EuroScore, median (IQR)c, ^δ^	5 (4–7)	5 (3–7)
Preoperative medication		
Statin	28 (93)	22 (69)
Βeta blocker	22 (73)	20 (63)
Diuretics	8 (27)	8 (25)
Aspirin	23 (77)	24 (75)
Calcium entry blockers	25 (83)	21 (66)
Nitrates	15 (50)	11 (34)
ACEI/ARBs	18 (60)	13 (41)
Valve surgery, ^ζ^	15 (50)	16 (50)
Duration of procedure, (SD), min	194 (18)	170 (10)
Duration of CPB, (SD), min	116 (12)	92 (7)
Duration of cross clamp time, (SD), min	85 (8)	70 (5)

Abbreviations: *ACEI*: ACE-inhibitors; *ARBs*: angiotensin II receptor blockers; *BSA*: body surface area; *CABG*: coronary artery bypass graft; *CPB*: cardiopulmonary bypass; *IQR*, interquartile range; *N*: number. *SI* conversion: To convert creatinine to mg/dL, multiply by 0.0113

^α^Data are shown as N (%) unless otherwise specified

^β^BSA calculated using the Haycock formula

^γ^ Definition of left ventricular function class: good, ejection fraction of >50 %, moderate, ejection fraction of 30–50 %; and poor, ejection fraction of <30 %

^δ^Higher EuroScores present increased risk of perioperative mortality

^ζ^Valve surgery includes aortic valve surgery

At the beginning of the operation, after induction of anaesthesia, echocardiographic data were collected in only 22 patients in each group. This is because preoperative ultrasound was not routinely assessed in all patients, as was the case with postoperative ultrasound. There were no differences in any of the obtained echocardiographic parameters between the dexamethasone group and the placebo group (Table 2). Also, no differences were found in preoperative and postoperative LA dimensions and function between patients undergoing valve surgery or CABG in both the placebo and dexamethasone group.

**Table 2:** Comparison of preoperative echocardiographic parameters in the dexamethasone and placebo groups (*N* = 44). Data shown in means ± SD

Ultrasound parameter	Dexamethasone (*N* = 22)	Placebo (*N* = 22)	Mean difference	95 % CI of difference	P Value
LA-TEF, %	41.1 (12.4)	42.2 (14.0)	−1.1	−9.2 to 6.9	0.77
LA diameter, cm	4.2 (0.7)	4.3 (0.8)	−0.1	−0.6 to 0.4	0.11
LA area, cm^2^	15.3 (5.0)	14.9 (4.6)	0.4	−2.4 to 3.4	0.75
LA max, ml	43.0 (19.6)	42.8 (18.6)	0.2	−11.4 to 11.9	0.97
LA min, ml	26.9 (16.2)	26.0 (15.2)	0.9	−8.6 to 10.5	0.85

Abbreviations: *CI*: confidence interval; *LA*: left atrial; *N*: number; *SD*: standard deviation; *TEF*: total ejection fraction

### Primary outcome

The mean LA-TEF after sternal closure was 36.4 % (±11.9 %) in the dexamethasone group and 40.2 % (±7.9 %) in the placebo group (mean difference −3.8 %; 95 % CI −9.0 to 1.4 %; *P* = 0.15) (Table 3).

**Table 3:** Comparison of postoperative echocardiographic parameters in the dexamethasone and placebo groups (*N* = 62). Data shown in means ± SD

Ultrasound parameter	Dexamethasone (*N* = 30)	Placebo (*N* = 32)	Mean difference	95 % CI of Difference	P Value
LA-TEF, %	36.4 (11.9)	40.2 (7.9)	−3.8	−9.0 to 1.4	0.15
LA diameter, cm	4.6 (0.8)	4.3 (0.9)	0.3	−0.2 to 0.7	0.19
LA area, cm^2^	16.0 (5.0)	16.4 (5.9)	−0.4	−3.1 to 2.4	0.81
LA max, ml	31.0 (12.3)	31.3 (13.9)	−0.3	−7.0 to 6.3	0.91
LA min, ml	19.7 (10.7)	18.8 (9.8)	0.9	−4.3 to 6.2	0.73
LAA area, cm^2^	2.9 (0.9)	2.7 (0.8)	0.2	−0.3 to 0.6	0.47
LAA velocity, cm/s	57.3 (19.6)	58.9 (16.3)	−1.6	−11.6 to 8.4	0.75
Left pulmonary vein S/D ratio	1.3 (0.2)	1.5 (0.4)	−0.2	−0.4 to 0.1	0.17

Abbreviations: *CI*: confidence interval; *LA*: left atrial; *LAA*: left atrial appendage; *N*: number; *S*/*D ratio*: systolic/diastolic ratio; *SD*: standard deviation; *TEF*: total ejection fraction

### Secondary outcomes

After sternal closure, there were no differences in LA diameter, LA area, maximum and minimum volume of the left atrium, LAA area and velocity, and left pulmonary S/D ratio between the two groups (Table 3).

PNAF occurred in 21 of the 62 patients (34 %) and was most commonly detected on the third postoperative day, with a median duration of1 day. In the dexamethasone group, nine patients developed PNAF (30 %), while 12 patients developed PNAF in the placebo group (39 %) (*P* = 0.47). In patients who developed PNAF, mean LA-TEF was 38.6 % (±9.1 %), as compared with 38.0 % (±10.9 %) in patients in whom PNAF did not occur (mean difference 0.6; 95 % CI: −4.6 to 5.9; *P* = 0.81).

## Discussion

In this study we evaluated the effect of high-dose intraoperative dexamethasone on echocardiographic measures of LA function, as a possible determinant of the risk for atrial fibrillation after cardiac surgery. No effect of dexamethasone on LA ejection fraction or dimensions was found when compared with placebo. Also, dexamethasone did not significantly reduce the risk of PNAF.

Atrial fibrillation is a very common cardiovascular disease with a lifetime risk in the general Dutch population of 25–30 % [21]. In the cardiac surgical population, its incidence is very high, being up to a third of all patients developing PNAF, and older age is still the only preoperative risk factor that has been consistently shown to predict the occurrence of PNAF [2, 3]. Nonetheless, LA dimensions and function have been implicated to be predictors of PNAF in the postoperative setting in several studies [9, 10]. Preoperative LA-TEF was shown by Haffajee et al. to predict PNAF even better than LA volume or diameter [10]. Osranek et al. found that preoperative maximum LA volume (32 ml/m^2^) was an important predictor of PNAF [9]. However, Gibson et al. did not confirm this [6].

The mechanisms that contribute to the development of LA dysfunction after cardiac surgery are still not entirely clear. In patients with a positive fluid balance, the left atrium is often exposed to a higher preload postoperatively when compared with the preoperative period. Additionally, due to surgical trauma, the left atrium is exposed to a systematic inflammatory response. The higher preload and the systemic inflammation may result in atrial dilatation and fibrotic infiltrates. These changes lead to temporary electromechanical and morphological changes in the atrial myocardium and can subsequently cause the formation of micro re-entrant pathways [14, 22]. Several anti-inflammatory drugs have been studied to reduce PNAF, e.g. statins and corticosteroids. Large observational studies and trials with statins for this indication have shown conflicting results, but in a recent systematic review it was concluded that statins have positive effects on protecting against PNAF [23–25]. In the current study the authors focused on investigating corticosteroids and their role in reducing PNAF. Corticosteroids are known to inhibit both inflammation and tissue changes in the heart, by suppressing inflammation-induced proliferation of myocardial fibroblasts as well as by preventing the activation of ERK1/2 and NF-κB [26, 27]. Several clinical studies have shown that intraoperative corticosteroids reduce the risk of PNAF and the consistency of these findings has recently been confirmed by several reviews and meta-analyses [17, 25, 28].

In this study, dexamethasone did not have any demonstrable effects on TEF, LA and LAA dimensions. These findings could be due to the fact that we have missed an actual effect of dexamethasone because the number of patients included in this study was relatively small. On the other hand, other factors could have contributed to missing an effect. The relatively high age of the study population (mean age of 70) could have contributed, since ageing often leads to a permanent loss of myocardial muscle fibres and an increase in fibrosis, resulting in irreversible atrial remodelling which may not be influenced by dexamethasone [29]. Also, LA dimensions and function strongly depend on loading conditions. Most patients are intravascularly well loaded after the operation and most are on inotropic support, which might have confounded the effect of dexamethasone on the left atrium. However, it seems very reasonable to assume that dexamethasone does not have an effect on LA-TEF at all. Moreover, no association between LA-TEF and PNAF could be demonstrated, and the incidence of PNAF was not reduced by dexamethasone. The latter is contradictory to several previous studies looking at the effects of steroids on PNAF, although it is in line with the findings of the DECS trial, where no effect of treatment on PNAF could be demonstrated in 4482 patients.

This study demonstrates that PNAF is a complex arrhythmia with multiple mechanisms underlying its pathology, whereby atrial remodelling through an inflammatory pathway may only play a minor role. Future studies should investigate atrial biopsies to attempt to elucidate subtle and delicate pathophysiological inflammatory and microfibrotic changes in the atrial myocardium, as well as the effects on this of anti-inflammatory agents.

### Strengths and limitations

The strengths of this study include the randomised double-blinded placebo-controlled design, which allowed for strict distribution of the patient’s clinical and demographic characteristics. Continuous Holter monitoring for 5 days postoperatively ensured that all PNAF episodes could be detected for accurate quantification.

A limitation of this study is that the sample size was small. Another limitation is that with transoesophageal echocardiography there might be an underestimation of LA dimension. As this holds for both study groups, and for the measurements obtained before and after surgery, we do not expect an effect on the study results obtained. Another limitation is the fact that LA-TEF was measured and calculated based on transoesophageal echocardiography images, using the same methods and mathematical formula that have been used in previous studies using transthoracic echocardiography. Although these methods have not been well validated in transoesophageal echocardiography, the potential error is probably the same for both groups.

## Conclusion

Intraoperative high-dose dexamethasone did not have any protective effect on postoperative LA-TEF or dimension and did not reduce the risk of PNAF in cardiac surgical patients.
